# Crucial Conversations for High-Risk Populations before Surgery: Advance Care Planning in a Preoperative Setting

**DOI:** 10.1089/pmr.2021.0015

**Published:** 2021-10-06

**Authors:** Roma Patel, Alexia Torke, Barb Nation, Ann Cottingham, Jennifer Hur, Rachel Gruber, Shilpee Sinha

**Affiliations:** ^1^ Indiana University School of Medicine, Indianapolis, Indiana, USA.; ^2^ IU Health Physicians, Indianapolis, Indiana, USA.; ^3^ Indiana University Center for Aging Research, Regenstrief Institute, Inc., Indianapolis, Indiana, USA.; ^4^ Division of General Internal Medicine and Geriatrics, School of Medicine, Indiana University, Indianapolis, Indiana, USA.; ^5^ Fairbanks Center for Medical Ethics, IU Health, Indianapolis, Indiana, USA.; ^6^ Daniel F. Evans Center for Spiritual and Religious Values in Healthcare, IU Health, Indianapolis, Indiana, USA.; ^7^ Advanced Scholars Program for Internists in Research and Education (ASPIRE) Indiana University (IU) School of Medicine, Indianapolis, Indiana, USA.; ^8^ Indiana University Center for Health Services and Outcomes Research, Regenstrief Institute, Inc., Indianapolis, Indiana, USA.

**Keywords:** advance care planning, mortality, preoperative clinic, readmissions

## Abstract

***Background:*** High-risk patients undergoing elective surgery are at risk for perioperative complications, including readmissions and death. Advance care planning (ACP) may allow for preparation for such events.

***Objectives:*** (1) To assess the completion rate of advance directives (ADs) and their association with one year readmissions and mortality (2) to examine clinical events for decedents.

***Design:*** This is an observational cohort study conducted through chart review.

***Setting/Subjects:*** Subjects were 400 patients undergoing preoperative evaluation for elective surgery at two hospitals in the United States.

***Measurements:*** The prevalence of ADs at the time of surgery and at one year, readmissions, and mortality at one year were determined.

***Results:*** Three-hundred ninety patients were included. In total, 102 (26.4%) patients were readmitted, yet did not complete an AD. Seventeen (4.4%) patients filed an AD during follow-up. Nineteen patients died and mortality rate was 4.9%. There was a significant association between completing an AD before death. Of the decedents, seven (37%) underwent resuscitation, but only four had ADs.

***Conclusions:*** Many high-risk surgical patients would benefit from ADs before clinical decline. Preoperative clinics present a missed opportunity to ensure ACP occurs before complications arise.

## Introduction

Chronic disease and frailty account for diminished cognitive and functional measures and are associated with high surgical risk and postoperative outcomes.^1–5^ High-risk patients who undergo elective surgery have an increased likelihood of life-threatening complications.^6–9^ In the perioperative period, patients often lack decision-making capacity, highlighting the importance of upstream advance care planning (ACP).^10,11^ ACP improves concordance between patient's wishes and medical interventions.^12,13^

There is emerging support for ACP in high-risk surgery patients,^10,14^ but the ideal timing of ACP discussions is not well defined.^15–17^ Counseling and documentation of advanced directives (ADs) are not standard in preoperative clinics, though research has demonstrated its feasability.^18^ Studies have shown completion of ADs is associated with lower readmission rates^19,20^ and decreased ICU utilization.^21^ This has implications for patients and health systems.^22–25^

We have previously published a retrospective chart review of patients seen in a preoperative clinic to identify the prevalence of ADs.^26^ In the present analysis, we followed these patients for one year. The primary objective was to assess the completion of ADs before surgery or in follow-up and association with readmissions or death at one year. The secondary objective was to examine the completion of ADs in decedents who required cardiopulmonary resuscitation and reintubation (defined as resuscitation).

## Methods

This study was an observational chart review cohort study conducted at two large urban academic hospitals. They are tertiary referral centers for the state's largest health care organization that totals 18 hospitals, >110,00 admissions per year, and >2500 beds. Four hundred consecutive patients between February and March 2017 referred for preoperative evaluation for elective surgery at two preadmission testing (PAT) clinics were identified. Patients are referred by their surgeons for risk assessment and optimization by internal medicine hospitalists who staff the PAT clinics. This study was approved by the Indiana University Institutional Review Board, which serves the entire organization.

Demographics and comorbidities were obtained from the preoperative evaluation. The electronic medical record (EMR) was reviewed at one year from the PAT clinic appointment for the presence of ADs, mortality, and readmissions. Patients were coded as having an AD if their EMR had a legal document naming an SDM (surrogate decision maker, including health care representative, health care power of attorney), and/or a living will, physicians order for scope of treatment or out of hospital do not resuscitate. Statistical methods included descriptive statistics of patient characteristics, and chi-square and Fisher's exact tests for comparison between groups.

## Results

A total of 400 charts were reviewed. At one year, 10 patients did not undergo surgery, leaving 390 records. The average age of patients was 58.4 years (standard deviation 14.6) and 187 (48%) were male. The cohort was predominantly white (85%). A previously published article discusses the preoperative risk, functional status, and comorbidities of this cohort.^26^ In brief, 87% of patients had an elevated perioperative risk of surgery, the mean revised Charlson comorbidity index was 1.9 (standard deviation 2.2), and 21% of patients had a functional capacity described as “borderline” or “poor.” Thirty percent of patients had cancer.

Only 63 (16.2%) patients had an AD in the EMR before surgery.^26^ Seventeen (4.4%) patients filed an AD during the follow-up period, 2 of whom had an AD on file at the time of surgery. There was no association between completion of AD with age, gender, or race (*p* > 0.05). One-hundred nineteen patients had cancer (Table 1). There was no statistically significant difference in survival or AD completion between those who had metastatic and nonmetastatic cancer. Of the seven patients who underwent palliative surgery, one had an AD before surgery and one completed an AD during follow-up.

**Table 1.** Characteristics of Patients with Cancer

Variable	Overall cohort, *N* = 390, *N* (%)	Nonmetastatic/unknown cancer, *N* = 93, *N* (%)	Metastatic cancer, *N* = 26, *N* (%)
AD on file before surgery	63 (16)	16 (17)	3 (14)
AD completed during follow-up	17 (4)	0 (0)	1 (5)
Palliative surgery	N/A	0 (0)	7 (27)
Survivors	371 (95)	82 (88)	22 (85)

ADs, advance directives.

In total, 102 (26.4%) patients were readmitted at least once and no association was found between AD completion and readmission (*p* = 0.42). About one-third of the readmissions were patients with cancer. An additional 40 (10.3%) patients returned to the emergency department but were not admitted.

**FIG. 1.**
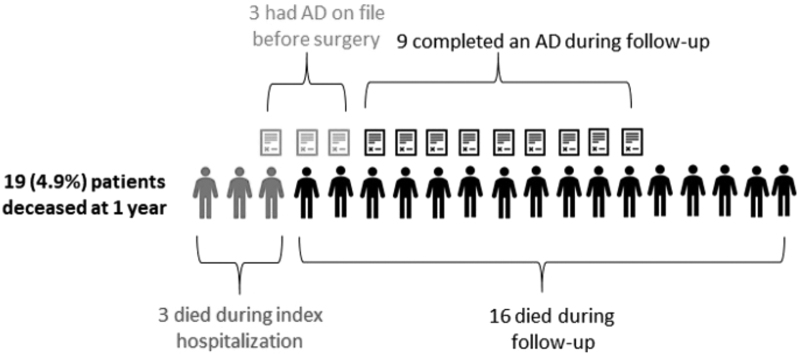
Mortality data.

Three patients died during the index hospitalization and 16 patients died during follow-up, for a total mortality rate of 4.9% (Table 2). Fourteen of the decedents had cancer. Four of them had metastatic cancer, none of whom had an AD on file before surgery. Twelve of the 19 (63%) decedents had an AD on file (Fig. 1), with 3 of them (16%) had an AD on file before surgery. Of the nine decedents who completed an AD during follow-up, two completed a living will, four completed an SDM document, and three completed both. Nine decedents completed an AD during follow-up (47%), compared with eight survivors (2%). Those with mortality had a strong correlation with AD completion (18% of survivors vs. 63% of decedents, *p* < 0.001).

**Table 2.** Characteristics and Outcomes of Survivors and Decedents

Variable	Survivors, *N* = 371, *N* (%)	Decedents, *N* = 19, *N* (%)
Gender		
Female	194 (52)	9 (47)
Surgery		
Cancer	102 (28)	14 (74)
Dental	16 (4)	2 (11)
General surgery	46 (12)	0 (0)
Ear, nose, and throat	34 (9)	0 (0)
Gynecology	11 (3)	0 (0)
Neurosurgery	61 (16)	0 (0)
Orthopedic	53 (14)	1 (5)
Urology	12 (3)	0 (0)
Other	36 (10)	2 (11)
Died during initial/surgical hospitalization	N/A	3 (16)
Died in the hospital	N/A	8 (42)
Resuscitation (CPR or intubation)	3 (1)	7 (37)
Died during code	N/A	2 (29)

Regarding our secondary objective, 10 patients (2.5%) underwent resuscitation during the follow-up period, 3 of whom survived. Of the surviving patients, one suffered a stroke and required reintubation, another required reintubation due to respiratory distress and ultimately required a tracheostomy; neither of these had an AD on file. The third patient required reintubation and vasopressors but had a living will on file and was able to express desire for reintubation to the medical team. Of the 19 patients who died, 7 (37%) underwent resuscitation—3 of whom were intubated and 4 of whom experienced cardiopulmonary arrest. The five patients had return of spontaneous circulation but were ultimately transitioned to comfort measures. Only four of the decedents who underwent resuscitation had an AD on file (two had an SDM and two had a living will). Both of the living wills stated generic language regarding their desire to die naturally without artificial prolongation of life if they were thought to have a terminal condition.

## Discussion

Similar to prior studies, a low proportion of our patients had an AD on file.^27,28^ Patients with metastatic cancer did not differ in their rate of AD completion. There was a low rate of palliative surgery, likely due to selection bias of patients sent to the PAT clinic. This may be due to an understanding that these patients may not be able to be medically optimized, and the risks of surgical intervention outweigh the benefits. Over a quarter of the patients were readmitted during follow-up, which is a large proportion, though in line with prior studies.^29^ In contrast to prior studies, our data did not find an association between having an AD on file and reduced likelihood of readmission.^19,20^ Readmissions still highlight a missed opportunity as this cohort has more contacts with the health system and are potentially sicker.

Our one-year mortality rate was <5%. In-hospital and/or six-month mortality rates of patients undergoing elective surgeries were between <1% and 3%.^1,2,30^ Although there was a statistically significant relationship between death and completing a prior AD, some were in response to the patient's imminently declining condition, emphasized by 47% of decedents completing an AD compared with 2% of survivors. Similar AD completion during clinical decline was seen in a prior study.^27^

Although the ideal time to complete ADs is poorly defined, early engagement in ACP discussions is desired and recommended.^31–33^ There are various barriers including time constraints, patient and disease characteristics, concern about receptivity, provider comfort and skill, disease trajectory, and prognostic uncertainty.^15–17,34^ Change in patient's clinical condition, hospital admission, and surgery have been identified as possible key trigger points for ACP discussion.^10,14,16,17,34^ Although it may be appropriate for patients to complete ADs as they become seriously ill, these findings suggest a missed opportunity for identifying higher risk patients early in the trajectory and facilitate goals-of-care conversations and complete ADs in advance. The preoperative clinic setting provides one touch point with the health care system with a cohort of identifiable high-risk patients and a potential target to initiate ACP discussions to reduce the proportion of patients who die or undergo resuscitation without prior ACP.

There are several limitations to this study. It was conducted within one health system and tertiary care hospitals, which may attract a higher risk surgical population. Observational data were collected over only one year. In addition, there is likely a selection bias among the patients as those who were sent to the PAT for evaluation as this was based on the surgeon's discretion. Moreover, this study did not examine the contents of the ACP documents nor discussions regarding goals of care to assess for concordance of readmissions and/or resuscitation with patient's wishes.

## Conclusion

One year follow-up of a high-risk surgical population found more decedents completed an AD compared with survivors, but the overall number of decedents who had completed an AD was still low and done late in the trajectory of illness. This highlights an opportunity for earlier timed ACP discussions. Our finding that postoperative resuscitation efforts were usually performed when there was no AD further supports the need for more studies aimed at optimal timing for ACP discussions, AD completion, and opportunities preoperative clinics provide for high-risk patients undergoing surgical interventions for ACP discussions earlier in their clinical trajectory.

## Abbreviations Used

ACP: advance care planning
ADs: advance directives
EMR: electronic medical record
PAT: preadmission testing
SDM: surrogate decision maker

## References

[1] Berry AJ, Smith RB, Weintraub WS, et al.: Age versus comorbidities as risk factors for complications after elective abdominal aortic reconstructive surgery. J Vasc Surg 2001;33:345–352. 11174788 10.1067/mva.2001.111737

[2] Naughton C, Feneck RO: The impact of age on 6-month survival in patients with cardiovascular risk factors undergoing elective non-cardiac surgery. Int J Clin Pract 2007;61:768–776. 17493090 10.1111/j.1742-1241.2007.01304.x

[3] Mrdutt MM, Papaconstantinou HT, Robinson BD, et al.: Preoperative frailty and surgical outcomes across diverse surgical subspecialties in a large health care system. J Am Coll Surg 2019;228:482–490. 30885474 10.1016/j.jamcollsurg.2018.12.036

[4] Khan KT, Hemati K, Donovan AL: Geriatric physiology and the frailty syndrome. Anesthesiol Clin 2019;37:453–474. 31337478 10.1016/j.anclin.2019.04.006

[5] McIsaac DI, Taljaard M, Bryson GL, et al.: Frailty as a predictor of death or new disability after surgery. Ann Surg 2020;271:283–289. 30048320 10.1097/SLA.0000000000002967

[6] Payá-Llorente C, Martínez-López E, Sebastián-Tomás JC, et al.: The impact of age and comorbidity on the postoperative outcomes after emergency surgical management of complicated intra-abdominal infections. Sci Rep 2020;10:1631. 32005885 10.1038/s41598-020-58453-1 PMC6994579

[7] Berian JR, Mohanty S, Ko CY, et al.: Association of loss of independence with readmission and death after discharge in older patients after surgical procedures. JAMA Surg 2016;151:e161689. 27409710 10.1001/jamasurg.2016.1689

[8] Lee DH, Buth KJ, Martin BJ, et al.: Frail patients are at increased risk for mortality and prolonged institutional care after cardiac surgery. Circulation 2010;121:973–978. 20159833 10.1161/CIRCULATIONAHA.108.841437

[9] Nelson MT, Spencer CC, Thompson A: 2014 ACC/AHA guideline on perioperative cardiovascular evaluation and management of patients undergoing noncardiac surgery 2014;64. [Epub ahead of print]; DOI: 10.1016/j.jacc.2014.07.944.

[10] Blackwood DH, Vindrola-Padros C, Mythen MG, Walker D: Advance-care-planning and end-of-life discussions in the perioperative period: A review of healthcare professionals' knowledge, attitudes, and training. Br J Anaesth 2018;121:1138–1147. 30336859 10.1016/j.bja.2018.05.075

[11] Silvester W, Detering K: Advance directives, perioperative care and end-of-life planning. Best Pract Res Clin Anaesthesiol 2011;25:451–460. 21925409 10.1016/j.bpa.2011.07.005

[12] Detering KM, Hancock AD, Reade MC, Silvester W: The impact of advance care planning on end of life care in elderly patients: Randomised controlled trial. BMJ 2010;340:847. 10.1136/bmj.c1345 PMC2844949 20332506

[13] Brinkman-stoppelenburg A, Rietjens JAC, Heide A Van Der: The effects of advance care planning on end-of-life care: A systematic review. Palliat Med 2014;28:1000–1025. 24651708 10.1177/0269216314526272

[14] Schuster ALR, Aslakson RA, Bridges JFP: Creating an advance-care-planning decision aid for high-risk surgery: A qualitative study. BMC Palliat Care 2014;13:1–8. 25067908 10.1186/1472-684X-13-32 PMC4110535

[15] Van Der Steen JT, Van Soest-Poortvliet MC, Onwuteaka-Philipsen BD, et al.: Factors associated with initiation of advance care planning in dementia: A systematic review. J Alzheimer's Dis 2014;40:743–757. 24531163 10.3233/JAD-131967

[16] Meehan E, Foley T, Kelly C, et al.: Advance care planning for individuals with chronic obstructive pulmonary disease: A scoping review of the literature. J Pain Symptom Manage 2020;59:1344–1361. 31837455 10.1016/j.jpainsymman.2019.12.010

[17] Mohan D, Sacks OA, O'Malley J, et al.: A new standard for advance care planning (ACP) conversations in the hospital: Results from a Delphi panel. J Gen Intern Med 2021;36:69–76. 32816240 10.1007/s11606-020-06150-0 PMC7859119

[18] Grimaldo DA, Wiener-kronish JP, Jurson T, et al.: A randomized, controlled trial of advance care planning discussions during preoperative evaluations. Anesthesiology 2001;95:43–50. 11465582 10.1097/00000542-200107000-00012

[19] J M, BA S: Integration of an advance care planning model in home health: Favorable outcomes in end-of-life discussions, POLST rates, and 60-day hospital readmissions. Home Heal Now 2019;37:337–344. 10.1097/NHH.0000000000000797 31688468

[20] Bond WF, Kim M, Franciskovich CM, Al BET: Advance care planning in an accountable care organization is associated with increased advanced directive documentation and decreased costs. J Palliat Med 2018;21. [Epub ahead of print]; DOI: 10.1089/jpm.2017.0566. PMC5867515 29206564

[21] Khandelwal N, Kross E, Engelberg R, et al.: Estimating the effect of palliative care interventions and advance care planning on ICU utilization: A systematic review. Crit Care Med 2015;43:1102–1111. 25574794 10.1097/CCM.0000000000000852 PMC4499326

[22] Flaatten H, De Lange DW, Morandi A, et al.: The impact of frailty on ICU and 30-day mortality and the level of care in very elderly patients (≥ 80 years). Intensive Care Med 2017;43:1820–1828. 28936626 10.1007/s00134-017-4940-8

[23] Glance LG, Kellermann AL, Osler TM, et al.: Hospital readmission after noncardiac surgery: The role of major complications. JAMA Surg 2014;149:439–445. 24599504 10.1001/jamasurg.2014.4

[24] Morris MS, Deierhoi RJ, Richman JS, et al.: The relationship between timing of surgical complications and hospital readmission. JAMA Surg 2014;149:348–354. 24522747 10.1001/jamasurg.2013.4064

[25] Merkow RP, Ju MH, Chung JW, et al.: Underlying reasons associated with hospital readmission following surgery in the United States. JAMA 2015;313:483–495. 25647204 10.1001/jama.2014.18614

[26] Sinha S, Gruber RN, Cottingham AH, et al.: Advance care planning in a preoperative clinic: A retrospective chart review. J Gen Intern Med 2019;34:523–525. 30604122 10.1007/s11606-018-4744-8 PMC6445838

[27] Marcia L, Ashman ZW, Pillado EB, Kim DY, Plurad DS: Advance directive and do-not-resuscitate status among advanced cancer patients with acute care surgical consultation. Armenean Surg 2018;84:1565–1569. 10.1177/000313481808401005 30747670

[28] Gamertsfelder EM, Seaman JB, Tate J, et al.: Prevalence of advance directives among older adults admitted to intensive care units and requiring mechanical ventilation. J Gerontol Nurs 2016;42:34–41. 10.3928/00989134-20151124-02 PMC6345507 26651862

[29] Jencks SF, Williams MV, Coleman EA: Rehospitalizations among patients in the Medicare Fee-for-Service Program. N Engl J Med 2009;360:1418–1428. 19339721 10.1056/NEJMsa0803563

[30] Blitz JD, Kendale SM, Jain SK, et al.: Preoperative evaluation clinic visit is associated with decreased risk of in-hospital postoperative mortality. Anesthes 2016;125:280–294. 10.1097/ALN.0000000000001193 27433746

[31] Crosby EM, Gutierrez KM: Facilitating timely advance care planning: Discussions in the ambulatory setting with a heart failure population. J Hosp Palliat Nurs 2019;21:445–452. 31425316 10.1097/NJH.0000000000000588

[32] Mechler K, Liantonio J: Palliative care approach to chronic diseases: End stages of heart failure, chronic obstructive pulmonary disease, liver failure, and renal failure. Prim Care 2019;46:415–432. 31375190 10.1016/j.pop.2019.05.008

[33] Kubi B, Istl AC, Lee KT, et al.: Advance care planning in cancer: Patient preferences for personnel and timing. JCO Oncol Pract 2020;16:e875–e883. 32282265 10.1200/JOP.19.00367

[34] Schichtel M, Wee B, Perera R, Onakpoya I: The effect of advance care planning on heart failure: A systematic review and meta-analysis. J Gen Intern Med 2020;35:874–884. 31720968 10.1007/s11606-019-05482-w PMC7080664

